# The impact of self-reported life stress on current impulsivity in cocaine dependent adults

**DOI:** 10.1016/j.pnpbp.2013.06.002

**Published:** 2013-06-21

**Authors:** Elizabeth L. Ross, Jin H. Yoon, James J. Mahoney, Yasmine Omar, Thomas F. Newton, Richard De La Garza

**Affiliations:** a The University of Houston, Department of Psychology, United States; b Baylor College of Medicine, Menninger Department of Psychiatry and Behavioral Sciences, United States

**Keywords:** Addiction, Cocaine, Dependence, Impulsivity, Life stress

## Abstract

Current cocaine treatments may be enhanced with a better understanding of the underlying mechanisms that contribute to the onset and maintenance of the disease, such as life stress and impulsivity. Life stress and impulsivity have previously been studied independently as contributors to drug use, and the current study expands upon past research by examining how these factors interact with one another. The aim of the current study was to evaluate the role of life stress in predicting impulsivity in a non-treatment seeking cocaine-dependent sample (*N* = 112). Analyses revealed that trait impulsivity (as measured by the Barratt Impulsiveness Scale) was associated with education (*r* = −3.09, *p* < 0.01), as those who had higher educational attainment also reported lower rates of trait impulsivity. In addition, those over the age of 30 demonstrated lower impulsivity in decision-making (as measured by delay discounting) than those under 30 (*t* = 2.21, *p* = 0.03). Overall exposure to life stress was not significantly correlated to either aspect of impulsivity. However several specific life stressors were significantly related to greater impulsivity including having been put up for adoption or in foster care (*t* = −2.96, *p* < 0.01), and having a child taken away against their will (*t* = −2.68, *p* = 0.01). These findings suggest that age and education relate to impulsivity; and that while an overall compilation of life stress scores was not related to impulsivity, specific types of stress related to either being taken away from a parent or having a child taken away were. Future studies should assess these constructs longitudinally to restrict response bias.

## 1. Introduction

Cocaine-dependence is a significant public health concern and according to the most recent National Drug Use and Health survey, 1.4 million U.S. residents are cocaine-dependent (Substance Abuse and Mental Health Services Administration Public Health Service U.S. Department of Health and Human Services, 2010). Cocaine-dependence is associated with a number of serious risks including: health problems, increased mortality, overdose, neonatal exposure to drugs, incarceration, violence, unemployment, and homelessness (Karch, 2005; Kruszon-Moran and McQuillan, 2005; Lucas, 2005; Nnadi et al., 2005; Schiller and Allen, 2005). Though national advocacy groups have increased drug prevention and treatment efforts, rates of use have only decreased slightly. Even among treatment seekers, relapse rates for drug use are high (Poling et al., 2007). Furthermore, the Food and Drug Administration has yet to approve any pharmacological interventions for cocaine-dependence. Thus, it is important to gain a better understanding of factors that contribute to cocaine-dependence in order to improve current treatments.

Two factors have consistently been identified as contributors to cocaine-dependence and use. One of these is exposure to life stress. Exposure to chronic stress (Felitti et al., 1998), and enduring repeated trauma that began early in life is associated with developing a substance use disorder (Lawson, 2013). In regards to cocaine, self-medication may be the result of strong relations between psychological stress and craving for cocaine (Sinha et al., 2000). In fact, a dose-dependent relation has been observed between the amount of exposure to lifetime stress and severity of cocaine use (Mahoney et al, 2012). These findings suggest that one reason people use substances is to cope with stressful life events.

Another key factor that is thought to play a large role in drug initiation and use is impulsivity (de Wit, 2009). Impulsivity may promote drug use in several ways. For one, drug users may prefer the immediate rewarding effects of drug consumption over the long-term benefits of abstaining, such as enhanced socio-economic status, diminished relationship conflict, and improved health (Kjome et al., 2010; Opris et al., 2009). Impulsive individuals also have more difficulty ignoring drug cravings (Tziortzis et al., 2011), as they tend to have impaired inhibitory restraint and attentional control (Liu et al., 2011; Prisciandaro et al., 2012). The contemporary view, supported by substantial empirical evidence, is that impulsivity represents a multi-dimensional construct with different measures capturing functionally different aspects of impulsivity (Evenden, 1999; Mitchell, 2004; Olmstead, 2006).

Though life stress and impulsivity have been studied as independent predictors of substance use in past studies; recent reports suggest that impulsivity and exposure to life stress interact with one another to create a generalized susceptibility to cocaine abuse and dependence (Hayaki et al., 2005). Furthermore, some researchers speculate that the role of environmental influences on the development of impulsive traits has previously been understated (Jakubczyk et al., 2013). They surmise that exposure to particular environmental circumstances earlier in life can influence adulthood impulsivity in decision-making, especially among individuals who have limited coping resources (Clarke and Schumann, 2009; Fishbein et al., 2006). Thus, the relationship between stress and level of addiction (Mahoney et al., 2012) may be partially explained by impulsivity (Hayaki et al., 2005); meaning that exposure to stress at an early age may contribute to the development of impulsive traits, and these traits predict greater drug use. However, among substance abusers, impulsivity is also related to: age (von Diemen et al., 2008), gender (Baker and Yardley, 2002), education (Loe and Feldman, 2007), and ethnicity (Jackson et al., 2007); and it is possible that these factors could account for the relation between prior life stress and impulsivity. The primary aim of the current study was to examine the role of life stress in predicting rates of impulsivity within a cocaine-dependent sample.

To our knowledge, only one study thus far has examined the relation between adversity and impulsivity in a drug-using sample. In that study, stress and impulsivity were significantly related above and beyond the influence of demographic variables, substance abuse and dependence, and number of substance-related diagnoses (Hayaki et al., 2005). These findings suggest that within a substance dependent population, exposure to stress and impulsivity are strongly related. However, this study only examined the role of recent stressors that occurred in the prior 6 months. The current study expanded upon these findings by including a measure of stress that covers the entire life span, and by utilizing multiple assessments of impulsivity. In the current study, we included both a measure of trait impulsivity, the Barratt Impulsiveness Scale (BIS; Patton et al., 1995) and a test of impulsive decision-making, delay discounting (DD; Mazur, 1987). Both tests have been associated with sustained cocaine use or the likelihood of relapse (Coffey et al., 2003; Heil et al., 2006; Moeller et al., 2002; Washio et al., 2011). Previous studies have not consistently reported that DD is significantly related to BIS scores, nor any particular subscale of the BIS (de Wit et al., 2007; Kirby et al., 1999; McLeish and Oxoby, 2007; Mobini et al., 2007), suggesting that these scales measure unique, non-related aspects of impulsivity. It was hypothesized that in this sample, life stress would significantly relate to both measures of impulsivity, after controlling for demographic characteristics. The goal of this study was to examine the interplay between impulsivity and exposure to life stress, factors that contribute to drug abuse, within a cocaine-dependent sample.

## 2. Methods

### 2.1. Participants

Participants included in this study were taken from a larger study that involved an inpatient hospital stay (e.g., Mahoney et al., 2012). For the current study, 112 participants recruited through newspaper and radio advertisements, who met criteria for cocaine-dependence according to the Mini-International Neuropsychiatry Interview (M.I.N.I.; Sheehan et al., 1998), and who had completed assessments of life stress and impulsivity were included in the analyses. Participants were excluded from the study if they met any of the following criteria: below 18 years of age, not currently using cocaine, treatment seeking, pregnant or nursing, current Axis I psychiatric disorder according to the M.I.N.I. Neuropsychiatric Interview, dependence on other drugs with the exception of nicotine, medical problems that would be affected by enrollment in the study (e.g., pre-existing heart condition), and if currently on probation or parole. Illicit drug use was also assessed via NIDA 5-Panel Drug Test Kits (Arham International, Inc., Greenville, SC), testing for cocaine, amphetamine, methamphetamine, THC, and opiate metabolites. In this study, participants reported using cocaine for 17 years on average. Participants typically used around 2 grams of cocaine per use, and reported using more days than not in the past month. Socio-demographic and drug-use characteristics are detailed in Table 1.

### 2.2. Procedure

This study was approved by both the Michael E. Debakey Veteran’s Affairs Medical Center (MDVAMC) Research and Development Committee and the Baylor College of Medicine Internal Review Board. Those who met criteria for study enrollment during the telephone screening were asked to come in for an in-person assessment at the Research Commons of the MEDVAMC. During this in-person assessment, participants were informed of the nature and purpose of the study, provided informed consent, and completed assessments relating to demographic information, drug use, impulsivity and life stress. Participants received a $40 gift card upon completion. After the in-person assessment, the research team determined if the individual was eligible to participate in one of the ongoing inpatient Phase I clinical trials. However, for this study, only the data from the initial in-person screening were included in the analyses; meaning that not all of the participants used in these analyses were enrolled in an inpatient clinical trial.

### 2.3. Measures

#### 2.3.1. Barratt Impulsiveness Scale

The Barratt Impulsiveness Scale (BIS; Patton et al., 1995) was selected to assess levels of trait impulsivity, as it has consistently been used to measure this construct in cocaine-dependent samples (Kjome et al., 2010; Liu et al., 2011; Schmitz et al., 2009). Each of the 30-items on the BIS is rated on a 4-point scale ranging from 1 (never) to 4 (always). Items on this scale are grouped into 3 higher-ordered factors: attentional impulsiveness, motor impulsiveness, and non-planning impulsiveness. These factors represent “acting without thinking,” “making quick cognitive decisions,” and “present orientation.” In the current study, the BIS total score was used in analysis of this trait. The BIS has sound psychometrics qualities as well, as internal consistency typically ranges from 0.79 to 0.83, and concurrent validity with other measures of impulsivity is high (Hanson et al., 2008). Moreover, within cocaine-dependent subjects, the BIS has been reliably used to measure trait impulsivity (e.g., Liu et al., 2011; Moeller et al., 2001).

#### 2.3.2. Delay discounting

In the current study, delay discounting (DD) was assessed using an adjusting amounts procedure (Mazur, 1987) via a computer-program running on Windows PC computer. Participants were presented with a choice between a relatively smaller, immediate option vs. $1000 after a fixed delay (e.g., “Would you rather have $500 now or $1000 1 year from now”). The value of the immediate option ranged between $0 and $1000 in $5 increments. The fixed delays were 1 day, 1 week, 1 month, 6 months, 1 year, 5 years, and 25 years. Delays were presented in an ascending or descending series and the order was counterbalanced across subjects. The value of the immediate, smaller option was designated by the program’s heuristic that utilized a titrating procedure to arrive at the point of subjective indifference. The DD score was assessed by fitting results to the hyperbolic equation: V=A/(1+kD) (Mazur, 1987), where V represents the subjective value of a reward of magnitude A presented at a delay of D. The value *k* within this equation measures the rate of discounting, and thus the amount of impulsive responding displayed by the participant. Higher *k* values represent greater discounting, which is a proxy for level of impulsivity in decision-making.

External validity of this measure has been demonstrated among drug users, as those with higher DD scores tend to engage in more high-risk behavior (Odum et al., 2000). Delay discounting has been widely used to measure impulsivity in decision-making within cocaine-dependent populations (Coffey et al., 2003; Heil et al., 2006). In fact, similar levels of discounting have been observed among actively using (in the past 2 weeks or month) and recently abstinent (in the past 2 weeks or month) cocaine-dependent individuals, suggesting that delay discounting may be relatively stable characteristic despite changes in drug use (Heil et al., 2006; Kirby and Petry, 2004; Washio et al., 2011).

#### 2.3.3. Life Stress Checklist — Revised

The Life Stress Checklist — Revised (LSC-R; Wolfe and Kimerling, 1997) measures the occurrence and severity of past trauma and/or stress. The LSC-R includes 30 events that could elicit post-traumatic stress symptoms such as: natural disasters, assault, abuse, and death of a friend or family member. Upon completion, the interviewer also asks the participant if any events occurred that were not mentioned among the 30 questions. For each item, the interviewer asks the participant whether or not the event occurred, the participant’s age(s) when the event occurred, the frequency of the occurrence, the participant’s perceived level of danger associated with the event, and the participant’s emotional reaction to the event. The LSC-R total score consists of the number of events endorsed. In this study, individual items were also included in the analyses, and given a score of 0 or 1 based on whether the individual reported that the event had occurred (Table 2).

Similar studies have indicated that this instrument has adequate test–retest reliability, with Kappa values ranging from 0.52 to 0.97 (McHugo et al., 2005). The LSC-R has demonstrated good concurrent validity with other measures of stress and trauma such as the Impact of Event Scale — Revised and the Symptom Checklist 90 — Revised, and high agreement with clinician ratings of trauma (Ungerer et al., 2010). Though not specifically intended for use in a cocaine-dependent population, this instrument has shown good criterion validity among individuals with dual diagnoses (McHugo et al., 2005), and has been used to detect high rates of PTSD comorbidity (Brown et al., 1999; Gil-Rivas et al., 2009). One study thus far (Mahoney et al., 2012) has utilized this instrument to assess stress and trauma in a cocaine-dependent sample; suggesting the LSC-R is a psychometrically sound instrument for assessing exposure to stress in this sample.

### 2.4. Data analytic strategy

#### 2.4.1. Preliminary analyses

Participants who were missing more than 50% of the data from a subscale or 80% of the data from a full scale were excluded from the analyses. In the current study, only 1 participant had to be excluded from the analyses for missing data. Preliminary analyses were conducted to determine whether the main study variables, life stress, impulsivity, or possible confounds were significantly related at the bivariate level. Independent sample *t*-tests and Pearson correlations were first conducted to evaluate whether demographic characteristics affected rates of life stress and impulsivity in this sample. The demographic variables of interest in this study included gender, age, education, and ethnicity. For age, participants were divided into two age groups: below 30 and above 30, due to fact that the portions of the brain associated with decision-making have reached full maturation by this age (Craik and Bialystok, 2006; Gong et al., 2009; Shaw et al., 2008). No other arbitrary categorical distinctions were made. Pearson Correlations were also used to assess bivariate relations between the main study variables: life stress (total life stress score and individual items from the LSC-R) and impulsivity (measured by both delay discounting and the BIS). At the bivariate level, the *p*-value was set at 0.05. Variables that were significantly correlated at the bivariate level were included in the multivariate analyses.

#### 2.4.2. Multivariate analyses

Multivariate analyses were performed to test the hypothesis that life stress would predict impulsivity after controlling for potential confounding variables. In these analyses, indices of life stress (total life stress and individual items from the LSC) would be entered into separate regression equations predicting impulsivity (measured by both DD and the BIS), dependent on whether these variables were related on a bivariate level. Confounds that were significant at the bivariate level were included as predictors in these equations as well. Because of the high number of analyses performed in this study, the *p*-value was set at 0.01 for multivariate analyses.

## 3. Results

### 3.1. Bivariate relations between demographic variables and rates of life stress and impulsivity

First, categorical demographic differences in rates of life stress and impulsivity were assessed using independent sample *t*-tests. There were no significant group differences in rates of life stress or impulsivity according to age or gender. However, when age was divided into those under 30 and those above 30, DD scores were significantly different between groups (*t* = 2.21, *p* = 0.03). Participants under the age of 30 (*M* = 5.51, *SD* = 24.28) had higher *K* scores on delay discounting than those over the age of 30 (*M* = 0.12, *SD* = 0.18). In addition, when assessed as a continuous variable, education was significantly correlated with BIS scores (*r* = −0.33, *p* < 0.01), as those with higher levels of education reported lower rates of trait impulsivity. A one-way ANOVA was used to assess ethnic differences in the main study variables, and revealed no significant differences.

### 3.2. Bivariate relations between life stress and impulsivity

In the current study, DD was not significantly correlated with the BIS total score (*r* = .06, *p* = 0.51), nor the BIS subscale scores: attentional impulsiveness (*r* = 0.08, *p* = 0.40), motor impulsiveness (*r* = −0.04, *p* = 0.70), and non-planning impulsiveness (*r* = 0.01, *p* = 0.30).

**Fig. 1:**
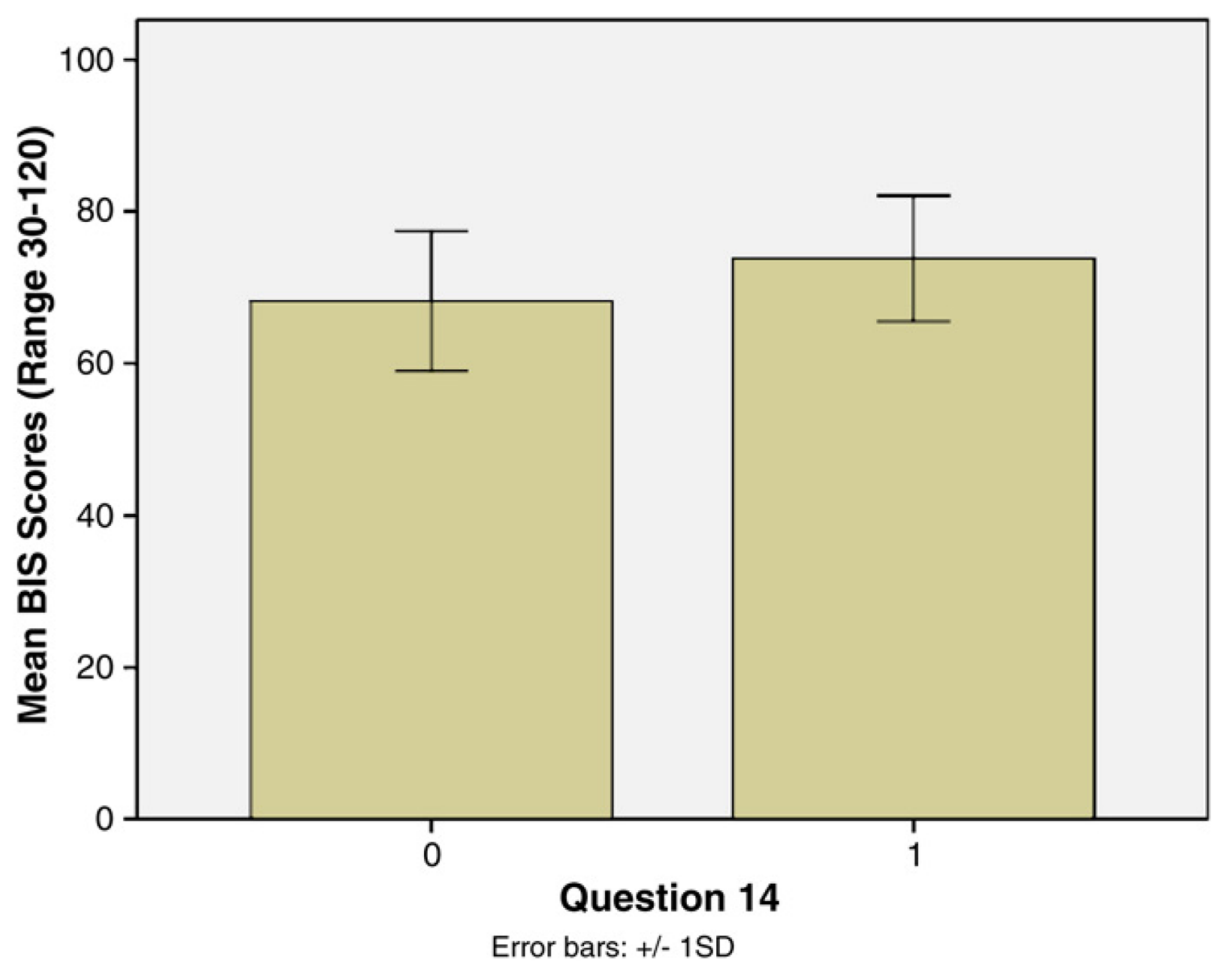
Mean BIS scores based on answers to Question 14 on the LSC-R regarding having a child taken away against one’s will.

**Fig. 2:**
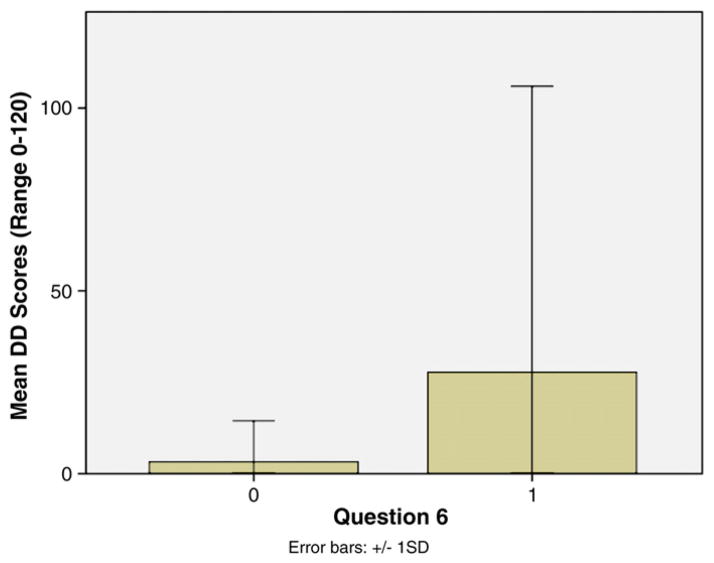
Mean DD scores based on answers to Question 6 on the LSC-R regarding having been put up for foster care or adoption.

In addition, neither the BIS (*r* = 0.02, *p* = 0.80), nor DD (*r* = −0.07, *p* = 0.48) were significantly correlated with total LSC-R scores (Table 3). T-tests were conducted with each of the items on the LSC-R on levels of impulsivity (BIS and DD) to determine whether experiencing a specific form of stress would impact the development of impulsive traits. Those who answered yes to Question 14 on the LSC-R relating to having a child taken away against their will also had higher total BIS scores (*t* = −2.68, *p* = 0.01); meaning that those who had their children taken away against their will had higher trait impulsivity (measured by the BIS) (*M* = 74.08, *SD* = 8.40) than those who had not had a child taken away (*M* = 68.67, *SD* = 10.01) (Fig. 1.). In addition, individuals who reported that they had been put in foster care or been put up for adoption themselves on Question 6 of the LSC-R scored significantly higher on delayed discounting (*M* = 27.71, *SD* = 78.19) than those who responded no to Question 6 (*M* = 3.24, *SD* = 11.21) (*t* = −2.96, *p* < 0.01); meaning those who did not grow up with their biological parents displayed more impulsivity in decision-making (Fig. 2.).

### 3.3. Life stress as a multivariate predictor of impulsivity

Since both Question 14 on the LSC-R regarding having a child taken away and level of education were related to BIS scores on the bivariate level, these variables were entered as multivariate predictors of BIS scores. The full model was significant, *F* = 10.41, *p* < 0.01, as was education, (*B* = −2.27, *SE* = .60, *β* = −0.33, *t* = −3.77, *p* < 0.01), and Question 14 on the LSC-R pertaining to having a child taken away, (*B* = 5.30 *SE* = 2.12, *β* = 0.22, *t* = 2.50, *p* = 0.01); suggesting that education and having a child taken away independently predict rates of trait impulsivity.

Next, the LSC-R adoption/foster care item was entered as a multivariate predictor of DD (measured by *k*), along with age, since both were significantly related to DD on the bivariate level. The full model was significant, *F* = 4.98, *p* < 0.01, as was the adoption/foster care item on the LSC, (*B* = 24.99, *SE* = 8.27, *β* = 0.28, *t* = 3.02, *p* < 0.01), while age was not, (*B* = −0.32, *SE* = 0.29, *β* = 0.10, *t* = 1.09, *p* = 0.28); suggesting that this form of life stress was a significant predictor of greater DD above and beyond the influence of age.

## 4. Discussion

The present study adds to a growing body of literature that has examined the roles of life stress and impulsivity among a substance-abusing population and expands upon previous studies by examining how these constructs interact with one another. In contrast to our hypotheses, total life stress score was not positively correlated with either measure of impulsivity. However, when individual life stress items were evaluated, significant differences emerged. Specifically, having a child taken away from you was significantly related to trait impulsivity, even after accounting for level of education. In addition, being put in foster care and/or up for adoption was significantly related to impulsivity in decision making after controlling for age. Though the primary aim of the study was to examine the role of life stress in explaining high rates of impulsivity in this population, analyses revealed that several demographic variables were also significantly related to impulsivity. Younger participants demonstrated higher rates of impulsivity in decision-making, while those with lower educational attainment displayed higher levels of trait impulsivity. These results suggest that a combination of demographic and environmental factors likely contribute to impulsivity and drug use among those who are cocaine dependent.

Given the reported significant relation between prior life stress and current impulsivity in past research (Hayaki et al., 2005), it was surprising that total life stress was not correlated with either measure of impulsivity. In interpreting these findings it is important to consider that the measures of impulsivity included in this study, while used previously in similar samples, did not assess every facet of impulsivity (e.g., disinhibition). Thus, it is possible that if a different measurement of impulsivity had been selected, these relations may have been statistically significant. In addition, low variability in rates of life stress and impulsivity in this study may in part account for the negative findings. This sample was fairly homogenous, and most of our participants scored in the higher range in measures of life stress (*M* = 8.13, *SD* = 3.81) and impulsivity (See Table 2). Because the study itself requires participants to stay at an inpatient facility for several days at a time, the majority of the participants in this study were unemployed and lived in low-income neighborhoods. For these reasons, it is likely that this particular group was more vulnerable to encountering stress, and lived in an environment where immediate survival may have been more pertinent to their day-to-day living than delayed rewards. Thus, it would be expected that this group of individuals would be more prone to high life stress and high levels of impulsivity. Above and beyond environmental influences, one cannot discount the impact of genetic factors on rates of impulsivity among substance abusers. According to a recent review on this subject (Verdejo-García et al., 2008), impulsive behavior among substance abusing groups has been linked to several specific genotypes. Thus, the impact of impulsivity on substance abuse is likely moderated by the presence of underlying genetic markers.

Though impulsivity did not relate to overall levels of life stress, two of the individual items on the LSC-R did significantly relate to indices of impulsivity. Those who responded yes to Question 14 on the LSC-R, which asked about having a child taken away against one’s will, also had significantly higher rates of trait impulsivity. Within the literature, there is also evidence of a relationship between impulsivity and inconsistency in parenting (Chen and Johnston, 2007). Since most individuals have children during adulthood, it is more likely that underlying impulsivity impacts the likelihood of this event occurring rather than vice versa. Thus, this finding suggests that impulsive people are more likely to have their children taken away, perhaps because they are more likely to make risky decisions that put their children in jeopardy of being harmed. In addition to the finding regarding the relation between trait impulsivity and having a child taken away, in this study findings suggested that being put up for adoption/or in foster care was significantly related to impulsivity in decision making. Likewise, children who have been ejected from the home of their biological parent and children who have been in multiple foster placements tend to display higher rates of impulsivity and distractibility (Linares et al., 2010). However, it is unclear whether this occurs because of a genetic predisposition to impulsivity, or if inconsistent parenting could impact an individual’s ability to self-regulate (Kinnally et al., 2009). Regardless, it appears that being raised by someone else other than your biological parent could serve as a particular risk factor for seeking immediate rather than delayed rewards. As noted earlier, among substance abusers, high impulsivity is particularly problematic as it can put these individuals at higher risk for relapse (Dallery and Raiff, 2007; Krishnan-Sarin et al., 2007; Washio et al., 2011; Yoon et al., 2007). Therefore, accounting for factors that contribute to impulsivity among this population could be important in prevention and treatment of substance abuse. Future studies should evaluate what underlying factors account for this relationship.

Lastly, the relations between one’s age and education and levels of impulsivity corroborated results from similar studies (e.g., Loe and Feldman, 2007). In the current study, those under the age of 30 displayed higher impulsivity in decision-making than those over the age of 30. It is thought that age is associated with impulsivity due to the fact that areas of the brain associated with decision-making and inhibition do not reach maturation until the mid-20’s (Craik and Bialystok, 2006; Gong et al., 2009; Shaw et al., 2008). In fact, other studies examining the impact of age on delay discounting have suggested that discounting rates decrease with age, but the relation between age and discounting levels off substantially after the age of 30 (Green et al., 1996; Yoon et al., 2007), suggesting this characteristic is fairly stable after that age. In addition, lack of educational attainment was related to trait impulsivity in the current study, consistent with the idea that highly impulsive people tend to seek more immediate gratification and are less likely to be motivated by the delayed gratification associated with obtaining an education (Spinella and Miley, 2004). In the literature it has been hypothesized that those with fewer years of schooling tend to minimize the consequences of continued drug use (Harder, 2007), and thus engage in a more impulsive decision-making process. Though not observed here, previous studies have reported a negative correlation between rates of discounting and education (Jaroni et al., 2004; Silva and Gross, 2004; Yoon et al., 2007). These results suggest that in addition to exposure to life stress, other environmental variables (age and education) are also associated with impulsivity among those who are cocaine dependent. Future research could examine these relations longitudinally to determine temporal precedence.

Though this study contributes to the growing body of literature examining factors that contribute to the onset and maintenance of substance abuse, several limitations should be conceded. The first is that along with interview-based measures, this research design relied on some self-report measures. Notwithstanding, self-reports have demonstrated reliability when confidentiality is ensured (Babor et al., 1990). Next, this study was cross-sectional in design. Due to this limitation, certain measures asked participants to recall information from the past that spanned a long period of time, and may have been subject to recall bias. Collecting data in a longitudinal design would allow us to better assess the true impact of predictor variables such as life stress on outcomes such as impulsivity. However, longitudinal data collection demands fiscal and clinical resources, and such a research design could only be implemented after supporting evidence has been collected. As noted earlier, the sample was a relatively homogeneous group, which may be expected in a substance abusing population. The sample was mostly male and middle-aged. Since males tend to display higher rates of impulsivity (e.g., Chapple and Johnson, 2007), and those who have lived longer have had more opportunities to encounter more major life stressors, as expected rates of impulsivity and life stress were negatively skewed in this sample. Selecting participants from differing backgrounds, or alternatively adding a control group of non substance-abusers, may increase variability and provide a more comprehensive picture of how life stress impacts impulsivity within a substance-abusing population. Finally, since some of the topics discussed in the LSC-R are sensitive and personal in nature, there is the possibility of participant’s under-reporting of their experiences. This under-reporting may not be a conscious decision by the participant, but rather a defense mechanism for coping with the traumatic event.

Despite these limitations, the current study is strengthened by several factors. The sample itself represented a fairly large group of individuals with severe cocaine-dependence, which enhances generalizability of the data. In this study, skilled technicians administered interview-based questionnaires, which allowed us to include clinically relevant information in our analyses. The measures we chose have been validated for this population, and were also well suited for the study aims. Moreover, the inclusion of two measures of impulsivity ensured that we have a more comprehensive picture of this construct. Taken together, this study provides additional insight into the relationship between prior life stress, impulsivity, and highlighted areas for future research within this population.

## Figures and Tables

**Table 1:** Sample demographics.

Variable	*N* (%)
Gender	
Male	93 (81%)
Female	22 (19%)
Ethnicity n (%)	
African American	90 (78.26%)
Caucasian	15 (13.04%)
Hispanic	7 (0.06%)
Other	3 (0.03%)
Age n (%)	
Above 30	101 (90.2%)
Below 30	11 (9.8%)
Education n (%)	
Graduated high school	93 (83%)
Below grade 12	18 (16.1%)
Method of cocaine use	
Smoke	105 (91.3%)
Intravenously	6 (0.05%)
Nasal	1 (0.01%)

**Table 2:** Descriptive statistics.

	Minimum	Maximum	Mean	Standard deviation
Life stress score	2	21	8.13	3.81
DD (LOG K)	0	221	4.98	23.29
BIS	47	94	69.83	9.91
Age	23	55	43.65	7.29
Education (years)	8	16	12.26	1.46

**Table 3:** Correlations between life stress, impulsivity, and demographic variables.

	Life stress score	DD (LOG K)	BIS	Age	Education (years)
Life stress score	1	–	–	–	–
DD (LOG K)	.04	1	–	–	–
BIS	.02	.06*	1	–	–
Age	−.16	.08	.02**	1	–
Education (years)	−.06	.02	−.33***	.15	1

Notes.

* *p* < .05.

** *p* < .01.

*** *p* < .001.

## Abbreviations

BIS: Barratt Impulsiveness Scale
DD: delay discounting
M.I.N.I.: Mini-International Neuropsychiatry Interview
LSC-R: life stress checklist-revised
